# Recent Advances in Functional Hydrogel for Repair of Abdominal Wall Defects: A Review

**DOI:** 10.34133/bmr.0031

**Published:** 2024-06-06

**Authors:** Ye Liu, Jinjian Huang, Sicheng Li, Ze Li, Canwen Chen, Guiwen Qu, Kang Chen, Yitian Teng, Rui Ma, Jianan Ren, Xiuwen Wu

**Affiliations:** ^1^ School of Medicine, Southeast University, Nanjing 210009, China.; ^2^ Research Institute of General Surgery, Jinling Hospital, Affiliated Hospital of Medical School, Nanjing University, Nanjing 210002, China.

## Abstract

The abdominal wall plays a crucial role in safeguarding the internal organs of the body, serving as an essential protective barrier. Defects in the abdominal wall are common due to surgery, infection, or trauma. Complex defects have limited self-healing capacity and require external intervention. Traditional treatments have drawbacks, and biomaterials have not fully achieved the desired outcomes. Hydrogel has emerged as a promising strategy that is extensively studied and applied in promoting tissue regeneration by filling or repairing damaged tissue due to its unique properties. This review summarizes the five prominent properties and advances in using hydrogels to enhance the healing and repair of abdominal wall defects: (a) good biocompatibility with host tissues that reduces adverse reactions and immune responses while supporting cell adhesion migration proliferation; (b) tunable mechanical properties matching those of the abdominal wall that adapt to normal movement deformations while reducing tissue stress, thereby influencing regulating cell behavior tissue regeneration; (c) drug carriers continuously delivering drugs and bioactive molecules to sites optimizing healing processes enhancing tissue regeneration; (d) promotion of cell interactions by simulating hydrated extracellular matrix environments, providing physical support, space, and cues for cell migration, adhesion, and proliferation; (e) easy manipulation and application in surgical procedures, allowing precise placement and close adhesion to the defective abdominal wall, providing mechanical support. Additionally, the advances of hydrogels for repairing defects in the abdominal wall are also mentioned. Finally, an overview is provided on the current obstacles and constraints faced by hydrogels, along with potential prospects in the repair of abdominal wall defects.

## Introduction

As a vital weight-bearing structure of the body, the abdominal wall functions critically in supporting respiratory mechanics and providing the necessary protective barrier for internal organs. It actively adapts to pressures within the abdomen and from the outside and is integral to normal physiological activities. Abdominal wall defects are characterized by the absence of one or more components of the abdominal wall. Most of these defects are acquired, with postoperative incisional hernias being the predominant kind, constituting more than 65% of all abdominal wall defects [1]. In addition, a variety of factors such as abdominal wall tumor resection, abdominal or intra-abdominal trauma, and infection can lead to severe and complex abdominal wall defects [2,3]. Abdominal wall defects have increasingly emerged as a prevalent clinical problem, impacting a substantial number of individuals globally. The worldwide occurrence rate of this ailment ranges from 9% to 20%. Annually, more than 400,000 reconstructive procedures are conducted to address interior soft tissue abnormalities, leading to healthcare expenses above 10 billion USD [4,5]. Apart from the primary manifestations of the underlying diseases, abdominal wall defects often cause functional impairments, including protrusion, decreased abdominal pressure, weakened respiratory function, gastrointestinal prolapse, and disrupted venous return. Furthermore, it is important not to overlook the potential repercussions that may arise from abdominal wall defects, including visceral herniation, intestinal obstruction, and intestinal adhesions [6].

Currently, there are a range of recommended treatments for abdominal wall defects, such as tension-free mesh repair [7], abdominal wall tissue separation [8], flap reconstruction [9], abdominal wall expansion techniques [10], and temporary abdominal closure measures [11,12]. While these technologies can partially reconstruct, restore, or compensate for lost function, they also expose inherent weaknesses. Complications such as infection and immune rejection, a high recurrence rate, and potential risks of reoperation prevent the establishment of advanced human functions and restrict their widespread clinical application [13–15]. Tissue engineering solutions offer novel approaches for enhancing the function of abdominal wall tissue through the integration of principles from engineering, life sciences, and materials science. These methods utilize scaffolds to transport cells as well as integrate biologically active substances that stimulate tissue repair and regeneration, functioning as a foundation for regenerative medicine [6,16–19]. Tissue engineering scaffolds are specifically engineered to provide structural support and create an optimal microenvironment that allows for the adhesion, migration, proliferation, and differentiation of cells [20]. Therefore, designing and fabricating an innovative bioscaffold is an important avenue for abdominal wall tissue engineering research to restore and reconstruct the structural and functional properties of abdominal wall tissues.

Hydrogel, composed of hydrophilic polymers arranged in a 3-dimensional structure, has attracted considerable interest as a possible solution. Hydrogels have several notable attributes that make them highly valuable in abdominal wall defect repair [21–23]. These include biocompatibility, the capacity to adjust mechanical properties, the capability to release drugs, the ability to mimic biological structures and interact with cells, and their user-friendly quality. Biocompatibility is a vital property of hydrogels, enabling their safe use in abdominal wall repair, facilitating tissue regeneration, and reducing the possibility of complications [24]. Hydrogels possess adjustable mechanical properties, which makes them extremely adaptable for reconstructing abdominal walls [25]. On one hand, they mimic the strength of abdominal wall tissue mechanics as well as offer the necessary mechanical reinforcement demanded during the repair procedure to prevent herniation and facilitate appropriate tissue remodeling [26]. Hydrogels can regulate the release of drugs or bioactive substances. This is especially beneficial in abdominal wall repair as it enables targeted administration of growth factors, anti-inflammatory agents, or antimicrobial agents. This promotes efficient healing and lowers the chances of postoperative infections [27]. Hydrogels exhibit biomimetic properties that allow them to mimic the biochemical and biomechanical cues present in the natural extracellular matrix (ECM). This imitation establishes a favorable microenvironment for cell attachment, growth, and specialization. It additionally encouraged neotissue formation while strengthening abdominal wall function and tissue structure [28,29]. Additionally, the outstanding level of user-friendliness displayed by hydrogels makes them exceedingly appealing for abdominal wall repair. This simplicity not only simplifies the surgical procedure when compared to traditional treatment methods but also enhances the practicality and implementation of hydrogel-based approaches in abdominal wall reconstruction [30–32]. Utilizing these properties will remarkably improve patient outcomes, promote tissue regeneration, decrease complications, and advance the field of abdominal wall restoration.

**Fig. 1.**
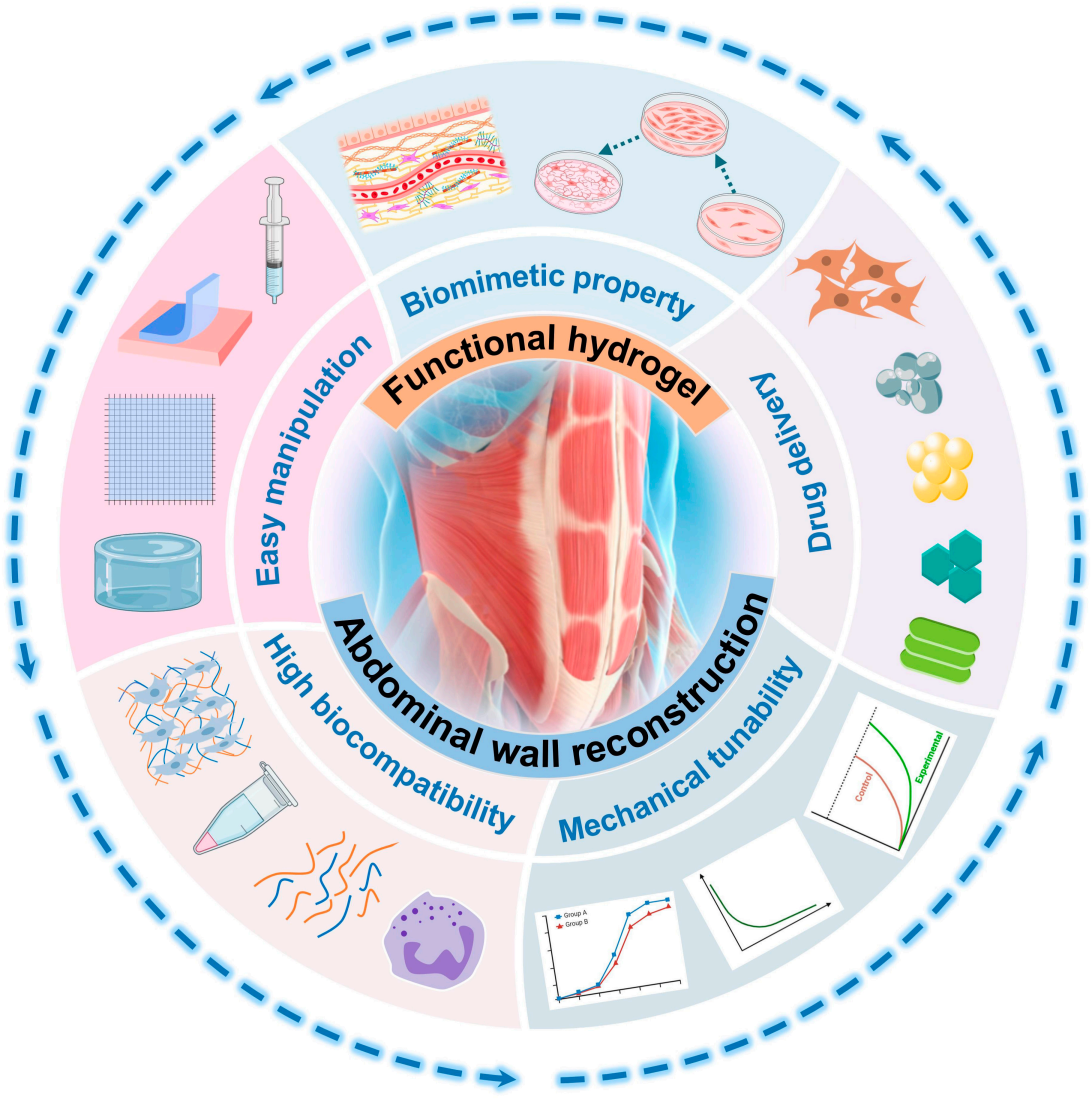
Illustrations of hydrogel with 5 prominent properties applied to repair abdominal wall defects.

This review aims to outline the five key benefits of hydrogels and their application in repairing and regenerating abdominal wall defects. It will discuss the advancements made in this field of research, providing insights into the design, development, and obstacles associated with hydrogel-based repairs for abdominal wall defects. Initially, we will present a concise overview of the arrangement and constitution of the abdominal wall, subsequently delving into an examination of the several categories of abdominal wall abnormalities and the presently accessible medical approaches for their treatment. Then, we will provide an in-depth explanation of the importance of the five main properties of hydrogels in repairing abdominal wall tissue. Additionally, we will emphasize the possible uses of hydrogels in fixing and regenerating abdominal wall defects. Then, we will examine and offer a perspective on the future advancements in repairing abdominal wall defects using hydrogel (Fig. 1).

## Abdominal Wall Anatomy and Available Treatments

### Abdominal wall tissue components and defects

The abdominal wall is composed of 6 layers: the skin, superficial fascia, muscles, transversalis fascia, preperitoneal fascia, and the peritoneum (Fig. S1A) [33]. It consists of the tissues located in the front and sides of the abdomen, including the region covered by the pair of rectus abdominis muscles and 3 layers of muscles in the lateral plane and adjacent to the spine. Besides protecting the organs in the abdominal cavity, the abdominal wall is also fundamental in keeping a straight spine, enabling bodily mobility, and aiding in ventilation, coughing, childbirth, urination, and defecation. The anterior abdominal wall has the dual role of protecting organs and stabilizing the placement of abdominal organs while maintaining a coordinated intra-abdominal pressure but is the most common site of abdominal wall injury [34].

Nevertheless, various congenital or acquired factors, such as tumor resection, tissue infection, and trauma can result in the occurrence of abdominal wall defects or injuries [35–39]. Abdominal wall defects are diverse and complex in terms of causes and types. To meet the needs of diagnosis and treatment, depending on the severity of abdominal wall defects, they can be divided into 3 different types: (a) Type I: Refers to cases where there is only the loss of skin and a portion of the subcutaneous tissue. (b) Type II: Mainly characterized by the absence of abdominal wall muscles and fascial tissues, while the skin remains intact. (c) Type III: The abdominal wall is completely absent. Both type II and type III abdominal wall defects usually require repair and reconstruction [18,19] (Fig. S1B).

Patients with abdominal wall defects frequently experience complications such as abdominal adhesions and intestinal obstruction, in addition to displaying clinical symptoms of the primary disease. These complications arise from multiple abdominal injuries and infections [40–42]. The procedure of forcefully separating adhesions might result in significant harm to the nearby intestinal tissues, ultimately resulting in the development of intestinal fistulas [43,44]. For example, patients with open abdomen frequently have many abdominal surgeries and repeated abdominal drainage, leading to recurring harm to the abdominal wall structure. This results in the formation of extensive and irregular defect areas, as well as complex defect kinds. In addition, prolonged bed rest, prolonged abdominal surgery, and lack of physical activity can lead to weakening and depletion of the abdominal wall muscles, making successful abdominal wall reconstruction difficult [45,46]. Hence, timely abdominal wall repair and reconstruction is crucial.

### Current treatments for abdominal wall repair

Nowadays, various approaches are available for the repair of abdominal wall defects (Table S1). The treatment of large and complex abdominal wall defects is more cumbersome compared to small and simple abdominal wall defects that can be directly sutured [47]. Artificial materials have undergone advancements and breakthroughs in repairing extensive abdominal wall defects in recent years [48]. Artificial muscle-skin flaps have been utilized to improve the repair of abdominal wall defects, significantly reduce the recurrence rate of different types of abdominal wall hernias, and reduce the occurrence of incisional hernias after large defects. However, tissue loss in the donor location is an unavoidable consequence of muscle flap transplantation. The surgical method is complicated, and there is a limited supply of tissue available for treating large abdominal wall defects [49,50]. Polypropylene (PP) mesh, which is a type of non-absorbable mesh patch, exhibits limited tissue biocompatibility and has the potential to induce significant tissue inflammation. This can result in the formation of abdominal adhesions and friction with the nearby intestine, ultimately leading to the development of intestinal fistulas [51]. Moreover, these materials do not possess inherent regeneration qualities. Although they can offer mechanical assistance, they do not stimulate tissue regeneration or improve cellular connections. Biological patches obtained from skin, small intestinal mucosa, or pericardium offer some benefits in terms of compatibility with living organisms, but they nevertheless exhibit elevated rates of hernia recurrence and unsatisfactory long-term repair results. In addition, individuals with wound infections, intra-abdominal infections, gastrointestinal fistulas, and immunological deficits are not appropriate candidates for the utilization of synthetic mesh [52–54]. Therefore, there is an urgent need for novel technology to tackle these challenges in clinical practice. The main purpose of abdominal wall repair is to ensure adequate mechanical support to protect the abdominal contents while obtaining a satisfied aesthetic appearance and true reconstruction of the abdominal wall.

## Hydrogel-Based Abdominal Wall Repair

Repairing and regenerating abdominal wall defects provide significant challenges. Hydrogels have arisen as a viable alternative to tackle these issues [55]. Currently, hydrogels commonly used for repairing abdominal wall defects are divided into 2 main categories, namely, natural hydrogels and synthetic hydrogels. Natural hydrogels are primarily based on collagen [56], fibrin glue [57], silk protein [58], and gelatin [59], which have advantages in terms of bioactivity. Synthetic hydrogels are mainly based on polyethylene glycol [60], polycaprolactone (PCL) [61], and polyvinyl alcohol (PVA) [62], which have advantages in mechanical properties. In addition, cross-linking is an essential factor in determining the properties and applications of hydrogels, and different cross-linking methods prepare hydrogels with different physicochemical properties and network structures. The cross-linking of hydrogels can be classified into physical cross-linking and chemical cross-linking [63]. Physical cross-linking is formed by molecular interactions, such as ion interactions, hydrogen bonding, and crystallization. Hydrogels formed by this method are usually reversible and possess advantages such as good biocompatibility and degradability. However, due to the generally weaker molecular interactions compared to chemical bonds, there are commonly issues of poor mechanical strength and insufficient stability. Chemical cross-linking involves covalent bond formation between polymer chains. Compared to physical cross-linking, hydrogels synthesized using chemical cross-linking methods hold better mechanical strength and stability but may pose potential cytotoxicity risks. Furthermore, depending on the specific requirements of abdominal wall repair applications, such as mechanical strength, degradation rate, and biological activity, different hydrogels and cross-linking methods can be chosen [64].

When hydrogels are used to treat abdominal wall defects, their natural biocompatibility greatly minimizes the occurrence of inflammatory reactions [65]. Modification of the hydrogel to match the mechanical strength, porosity, and degradation rate of the defect site can be achieved by adjusting the parameters such as polymer concentration, cross-linking mode, and degree of cross-linking. This allows them to provide suitable mechanical support and gradually degrade as the repair process advances, making space for the growth of new tissue [66]. Hydrogels are effective carriers of bioactive substances such as drugs, growth factors, and cells needed to repair abdominal wall tissues, providing convenient loading and accurate release. This release can be controlled based on external stimuli like light or pH [67]. Moreover, the inherent hydrophilicity of hydrogels creates a 3-dimensional environment very similar to the ECM. This environment facilitates cell adhesion, proliferation, and differentiation, while bioactive compounds can enhance cellular activity [68]. Crucially, hydrogels can be fabricated in diverse forms and can be introduced into the body through different methods, providing convenience and usefulness [69–71]. Moreover, hydrogels reveal exceptional ability to conform to various shapes and adhere to tissues, making them well-suited for irregularly shaped tissue defects. This allows for efficient and long-lasting repair at the site of the defect [72,73]. Thus, due to their distinctive features and adaptable abilities, hydrogels present intriguing opportunities for surpassing the constraints of conventional healing methods. As research advances, the benefits of these treatments become more and more evident, opening up fresh avenues for achieving effective abdominal wall repair.

### Five remarkable functional properties of hydrogels for defect abdominal wall repair

#### High biocompatibility with host tissues

Hydrogels are a unique class of materials that have attracted significant attention in the medical field due to their remarkable biocompatibility. This property allows them to be safely employed in a variety of medical applications, such as abdominal wall repair. The biocompatibility of hydrogels can be further divided into blood compatibility and tissue compatibility [74–76]. Due to the direct contact with blood, the assessment of blood compatibility is an important criterion for the successful application of hydrogels in abdominal wall repair. According to the guidelines established by the International Organization for Standardization (ISO) in 2000 (ISO 10993-4), the evaluation of blood compatibility involves 5 distinct categories: thrombosis, coagulation, platelets, hematology, and immunology (complement system and white blood cells) [77,78]. Yin et al. tested the blood biocompatibility of 3 different ratios of 4-arm-PEG-CHO/CMCS hydrogels. It demonstrated that all 3 hydrogel compositions had exceptional hemolysis resistance (Fig. S2A) with hemolysis rates significantly below 2% (Fig. S2B). This confirms the remarkable blood compatibility of 4-arm-PEG-CHO/CMCS hydrogels for abdominal wall defect patches [79]. The excellent blood biocompatibility of hydrogels provides favorable conditions for their success in applications of abdominal wall repair.

Previous studies indicated that hydrogels, unlike other materials used for repair, can interact with surrounding tissues beneficially when inserted into a living organism. This interaction reduces adverse reactions and immune responses, while also integrating well with the host tissues [80–82]. L929 fibroblasts were co-cultured with the JPVA hydrogel to evaluate their biocompatibility. Subsequently, cell viability staining (Fig. S2C) and CCK-8 assays (Fig. S2D) were performed. The cell morphology, cell density, and cell proliferation rate of the JPVA hydrogel group on days 1, 2, and 3 of culture were similar to those of the control group. Biocompatibility was further assessed in vivo via implantation of the hydrogel subcutaneously in the rat model for 5 days (Fig. S2E). Histologic analysis showed significantly lower inflammation levels in the JPVA hydrogel group compared to the PP mesh with the Parietex composite (PCO) mesh group (Fig. S2F). Additionally, immunohistochemical staining showed significantly lower CD68 (macrophage marker) (Fig. S2G) and interleukin-6 (one of the inflammatory factors) (Fig. S2H) expression in the JPVA hydrogel group compared to the PP mesh and PCO mesh groups [83]. It suggested that hydrogels have high biocompatibility and promote positive integration with host tissues. Studies are increasingly providing evidence for the utilization of hydrogels as an attractive option for repairing and regenerating abdominal wall defects.

#### Tunable mechanical properties matching the abdominal wall

**Fig. 2.**
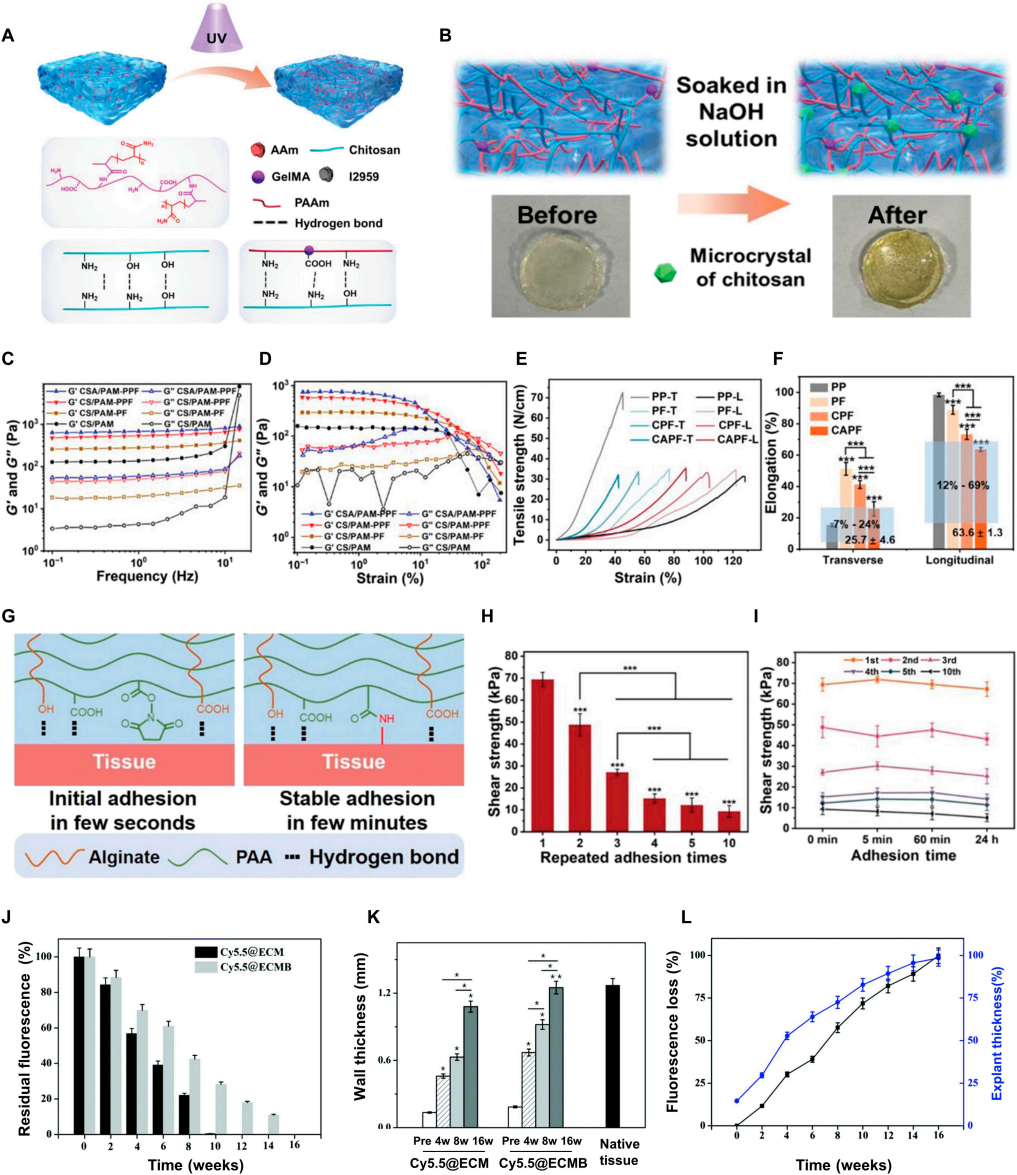
The excellent tunable mechanical properties of hydrogels. (A) Schematic of the CS/PAM hydrogel with molecular network prepared by UV treatment. (B) Schematic and photographs of the alkali treatment mechanism of CS/PAM and CSA/PAM hydrogels. (C) CS/PAM, CS/PAM- pf, CPF, and CAPF frequency scans and (D) strain sweep. (E) Commercial PP mesh, PF, CPF, and CAPF tensile strength–strain curves in transverse and longitudinal directions and (F) 16 N cm^−1^ elongation. (G) Mechanism of adhesion of CAPFAH hydrogels to wet tissue. (H) Hydrogel shear strength after multiple adhesion. (I) Adhesion strength of hydrogel with time after multiple adhesions. Reproduced from [93] with permission from John Wiley and Sons, Copyright 2023. (J) Cy5.5@ECM and Cy5.5@ECMB composites’ residual fluorescence. (K) Cy5.5@ECM and Cy5.5@ECMB composite explants’ wall thickness. (L) Cy5.5@ECMB composite fluorescence loss and explant thickness. Reproduced from [102] with permission from the Royal Society of Chemistry, Copyright 2021.

The abdominal wall is a dynamic structure that experiences various mechanical forces and continuous movement during daily activities. Hydrogels that match the mechanical properties of the abdominal wall are essential for the successful repair and regeneration of abdominal wall defects. Hydrogels possessing appropriate stiffness and elasticity tend to evenly distribute mechanical stress, alleviate strain on the healing tissue, and facilitate their integration with the surrounding natural tissue [84,85]. Moreover, matching the mechanical properties of the hydrogel to the abdominal wall also affects cell performance and tissue regeneration [86,87]. Cells are known to react to mechanical signals, and hydrogels are capable of simulating the mechanical environments observed in natural tissues. This mimicry allows cells to adhere, migrate, and differentiate, ultimately leading to enhanced tissue regeneration and functional recovery [88,89]. The mechanical properties of hydrogels depend mainly on their composition, cross-linking concentration, cross-linking technique, and polymer concentration [90–92]. Jiao et al. presented the preparation of a fabric hydrogel composite mesh, which mimics the mechanical properties of biological systems and has self-adhesive properties. For the hydrogel composite mesh, polyester knitted fabric and chitosan (CS)-polyacrylamide hydrogel composite were utilized. The hydrogel polymerization was increased by prolonging the photocross-linking period (Fig. 2A). Alkaline treatment resulted in physical cross-linking, creating small pores inside the hydrogel (Fig. 2B). The adhesive group boosted tissue compliance and significantly improved the rheological properties of the composite patches, maintaining stability in the physiological environment due to the hierarchical structure and mechanical anisotropy in the natural abdominal wall (Fig. 2C and D). Moreover, owing to the hydrogen bonding and topological entanglement, the hydrogel exhibited mechanical anisotropy and elasticity similar to abdominal wall tissues. The stresses in the horizontal and vertical directions were 25.7% ± 4.6% and 63.6% ± 1.3%, respectively, when subjected to a force of 16 N/cm (Fig. 2E and F). Furthermore, hydrogen bonding formed by alginate and PAA as well as covalent bonding formed by NHS esters with amino groups at the surface of the tissue resulted in both initial adhesion and stable long-term adhesion (Fig. 2G). This yielded a composite mesh characterized by high adhesion (70.1 ± 3.2 kPa) (Fig. 2H) with reproducible but robust adhesion (Fig. 2I) [93]. The simultaneous adaptability of both biomechanics and the mesh–tissue interface allows for the regeneration of the entire abdominal wall structure. Additionally, it offers valuable information on modifying the mechanical properties of hydrogels to correspond to the characteristics of the abdominal tissues. Moreover, the inclusion of nanoparticles, such as nanofibers, silver nanoparticles, hollow mesoporous silica particles, and other functional components, in the hydrogel matrix not only provides the hydrogel with several functions but also improves its mechanical properties even further [94–96]. These strategies can provide additional strength and structural support to the hydrogel, making it more suitable to repair abdominal wall defects.

Moreover, the degradation of the material is the key aspect to consider when matching hydrogel mechanical properties to the abdominal wall tissue regeneration process. Fluorescence imaging for monitoring material degradation has emerged as an innovative method for tracking the loss of fluorescent signals over time [97–101]. A study on real-time monitoring the material degradation and tissue remodeling utilized near-infrared fluorescent dye Cy5.5 NHS ester for labeling ECM composites (ECMB) performed from small intestinal submucosa (SIS) and CS/elastin electrostatically spun nanofibers. After implantation, the Cy5.5@ECMB composite material showed substantial fluorescence and had a degradation period of 16 weeks before the total breakdown occurred (Fig. 2J). The Cy5.5@ECMB composite implant thickness substantially grew at 4, 8, and 16 weeks after implantation, reaching a thickness similar to the normal abdominal wall at 16 weeks (Fig. 2K). Furthermore, the decrease in fluorescence was significantly and positively correlated with the thickness of the implanted Cy5.5@ECMB composite implant (*r* = 0.9832), suggesting a strong link between the degradation of the ECMB composite and tissue remodeling (Fig. 2L). Therefore, it not only achieved real-time monitoring of material degradation but also employed the thickness of the implant as an intuitive indicator for assessing the efficiency of tissue remodeling in ECMB composites, which provides new insights for evaluating and monitoring the tissue repair effectiveness of hydrogel [102].

#### Drug carriers for localized and controlled release

**Fig. 3.**
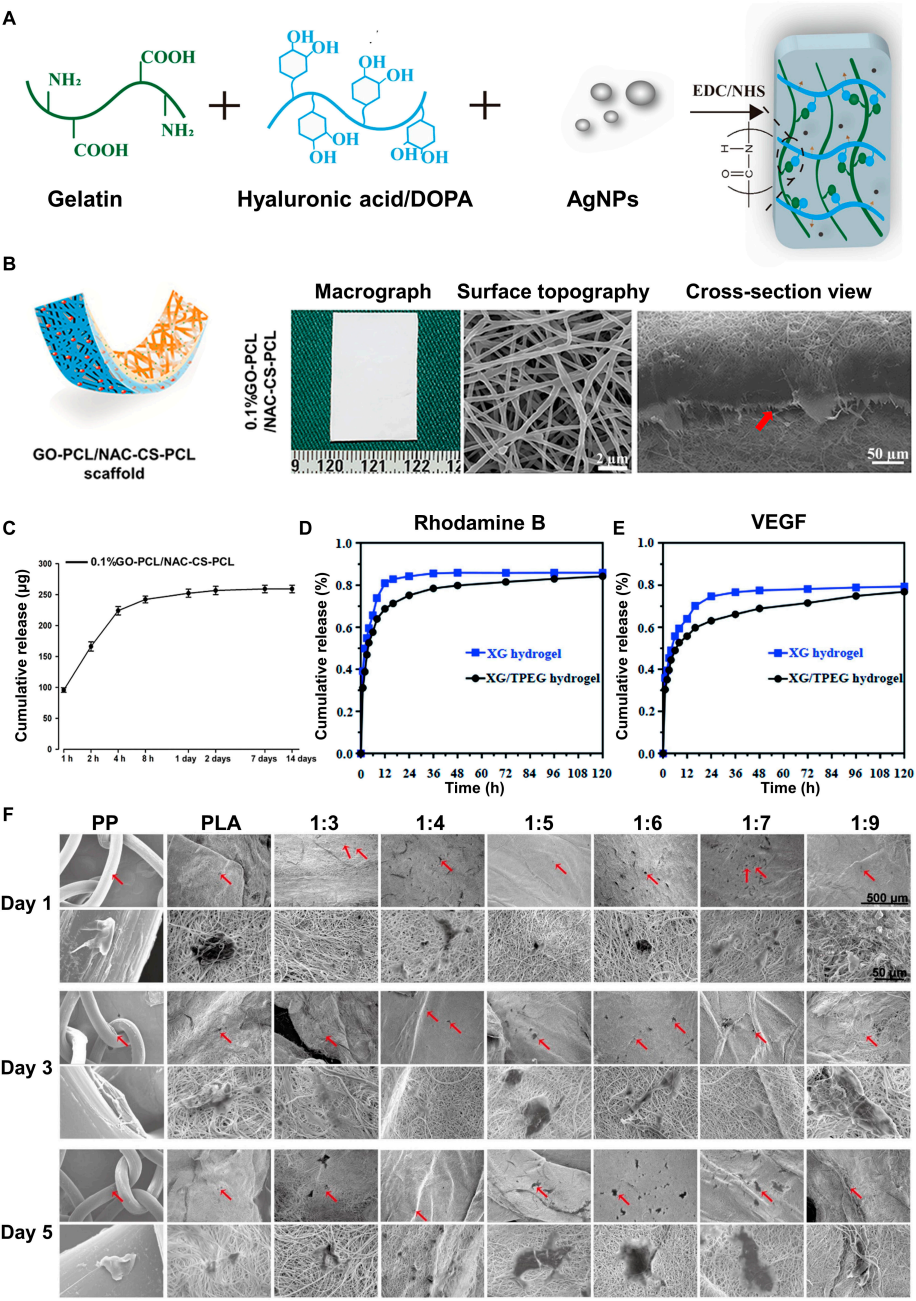
Drug carrier is one of the excellent properties of hydrogel. (A) A multifunctional hydrogel was synthesized for promoting abdominal wall defect repair based on dopamine-modified hyaluronic acid, gelatin, and nano-silver. Reproduced from [105] with permission from Elsevier, Copyright 2022. (B) An illustration, macrographs, and FESEM image of the GO-PCL/NAC-CS-PCL scaffolds. (C) NAC cumulative release from the scaffolds is time-dependent. Reproduced from [106] with permission from Dove Medical Press, Copyright 2021. (D) In vitro release pharmacological profile of Rhodamine B and (E) VEGF by simple XG hydrogel and XG/TPEG hydrogel carriers. Reproduced from [107] with permission from the Royal Society of Chemistry, Copyright 2020. (F) Scanning electron microscopy view of ADSCs (indicated by red arrows) cultured on scaffolds to show cell morphology. Reproduced from [108] with permission from Frontiers Media S.A, Copyright 2021.

Hydrogel can act as a drug carrier for regulated drug release in several biological applications, such as repairing abdominal wall defects [103]. Hydrogels, with their high water content, porous structure, and ability to retain drugs, enable the packaging, immobilization, and protection of small-molecule drugs, large-molecule drugs, and cells from denaturation. Hydrogels are capable of responding to internal and external stimuli, including light, pH, temperature, and redox triggers. It enables accurate regulation of the timing and location of drug release. Diverse approaches, such as physical and chemical procedures, are possibly used to integrate drugs into hydrogels. A prevalent approach for drug delivery is the direct integration of medicines or bioactive compounds into the hydrogel matrix during the manufacturing process. Wang et al. [104] synthesized a novel biomaterial consisting of submucosa and gelatin hydrogel for reconstructing abdominal wall defects, promoting better regeneration, and remodeling of host tissue by releasing alkaline fibroblast growth factor. Hu et al. created a dopamine-modified hyaluronic acid and gelatin hydrogel using an acylation process to combat severe infections in abdominal wall defects. It enhanced the antibacterial capabilities of the hydrogel via the addition of silver nanoparticles (Fig. 3A) [105]. Furthermore, drugs may be incorporated into hydrogels by utilizing the swelling feature of hydrogels, simply by immersing them in drug-containing fluids. Liu et al. described the fabrication of a bilayered nanofibrous membrane (GO-PCL/CS-PCL) utilizing continuous electrospinning. The membrane consists of PCL, CS, and graphene oxide (GO), as illustrated in Fig. 3B. To improve the biological capabilities of the patch (GO-PCL/NAC-CS-PCL) in promoting angiogenesis and reducing reactive oxygen species, *N*-acetylcysteine (NAC) was successfully incorporated into the fibrous membrane through immersion in a solution containing NAC (Fig. 3C) [106].

Hydrogels may regulate drug release by modifying the formulation, cross-linking density, or adding barriers or stimuli-responsive reactions. Li et al. discovered that both small-molecule drugs (e.g., Rhodamine B) (Fig. 3D) and large-molecule drugs (e.g., vascular endothelial growth factor [VEGF]) (Fig. 3E) exhibited significantly slower release rates in the interpenetrating network xanthan/4-arm polyethylene glycol thiol (XG/TPEG) hydrogel compared to pure XG hydrogel. Since the interpenetrating network formed by the XG/TPEG hydrogel creates a denser internal structure relative to the 3-dimensional (3D) network of the pure XG hydrogel, it limits the rapid release of the drug and prolongs its therapeutic effect. These interpenetrating dual-network hydrogels are highlighted for their advantages in tissue engineering and cytokine therapy [107].

Moreover, hydrogels are also favored as carriers for cell encapsulation because they may improve cell adherence and proliferation. Dong et al. incorporated the biodegradable polymer poly(lactic acid) (PLA) and poly(*N*-isopropylacrylamide)-b-poly(ethyleneglycol) (PNIPAAm-b-PEG) to create thermo-responsive hydrogel scaffolds utilizing electrostatic spinning technology. The composite electrospun scaffolds were inoculated with rat adipose-derived stem cells (ADSCs). Scanning electron microscopy (SEM) analysis revealed that the surface of various PNIPAAm-b-PEG/PLA scaffolds was adhered with more ADSCs spreading and stretching in a polygonal shape compared with PLA and PP scaffolds. It indicated that the PNIPAAm-b-PEG/PLA mimetic hydrogel scaffolds provided an improved surface for cell adhesion (Fig. 3F). These findings confirm the ability of hydrogels to facilitate an acceleration of cell adhesion and proliferation while efficiently loading cells [108].

Overall, hydrogels have promising potential as drug carriers for targeted delivery in abdominal wall defect repair. By incorporating drugs or other bioactive materials into the hydrogel matrix and achieving controlled release, hydrogels offer an appealing way to accelerate the repair of abdominal wall defects while reducing systemic side effects.

#### Biomimetic ECM to facilitate cellular interactions

**Fig. 4.**
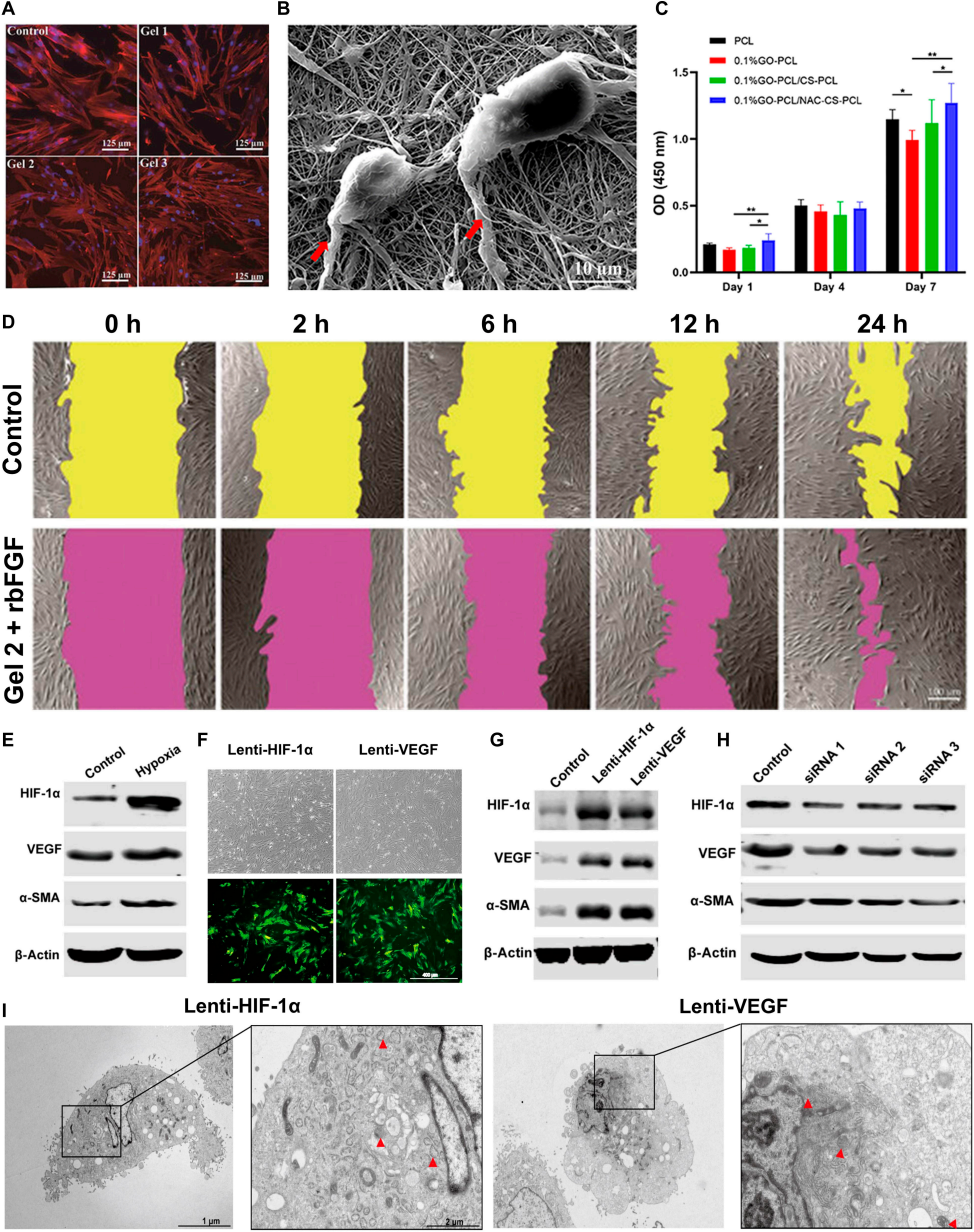
Biomimetic property is one of the excellent properties of hydrogel. (A) hGFs co-cultured with Gel 1 to 3 fluorescence images. Reproduced from [79] with permission from John Wiley and Sons, Copyright 2021. (B) Morphology of human dermal fibroblasts cultured on 0.1% GO-PCL/NAC-CS-PCL scaffold. Cell pseudopods are denoted by red arrows. (C) CCK-8 assay of human dermal fibroblasts. Reproduced from [106] with permission from Dove Medical Press, Copyright 2021. (D) Scratches healing experiments with images of hGF migration at different times. Reproduced from [79] with permission from John Wiley and Sons, Copyright 2021. (E) Western blot to examine the expression of HIF-1α, VEGF, and α-SMA in hypoxia-induced ADSCs. (F) Transfection of ADSCs with lenti-HIF-1α and lenti-VEGF for 72 h. (G) Western blotting to examine the expression and analysis of HIF-1α, VEGF, and α-SMA in lenti-HIF-1α and lenti-VEGF-transfected ADSCs. (H) Western blot to examine the expression and analysis of HIF-1α, VEGF, and α-SMA after HIF-1α siRNA knockdown of ADSCs. (I) WPBs (red triangles) were visible by TEM after transfection of lenti-HIF-1α and lenti-VEGF with ADSCs. Reproduced from [108] with permission from Frontiers Media S.A, Copyright 2021.

The ECM is a complex network of macromolecules secreted by cells with multiple functions [109]. It not only provides structural support and a microenvironment for the survival of tissue cells, but also promotes intercellular communication, regulates cellular behavior and immune responses, and stores and regulates growth factors as well as other bioactive molecules. These functions greatly influence the mechanical and physiological characteristics of tissue cells [110–113]. Hydrogels, due to their unique properties, are widely regarded as the preferred biomaterials for mimicking the natural ECM. They provide physical support, spatial organization, and cues for cell migration, adhesion, and proliferation [114]. To begin with, the hydrophilicity of hydrogels allows them to absorb and retain water, creating a hydrated environment. This hydration facilitates cell survival, allows the spread of nutrients and oxygen, and stimulates cellular functions. Furthermore, hydrogels enable the exchange of growth factors, cytokines, and other signaling molecules that are essential for cellular communication and tissue regeneration due to their porous structure. The porous hydrogel also permits the diffusion of nutrients, oxygen, and other essential molecules needed for cell survival and growth. Most importantly, hydrogel scaffolds provide a physical framework for cell migration, proliferation, and tissue formation. Cells can migrate into the hydrogel and fill its structure, resulting in the creation of new tissue. For example, hydrogels provide a favored microenvironment in which human gingival fibroblasts grow and adhere, leading to elongated cell formation, increased proliferation, and spreading (Fig. 4A) [79]. Consequently, hydrogels are thought to replicate the natural ECM, creating an ideal microenvironment for cell infiltration and growth in abdominal wall defects [115].

Meanwhile, hydrogels can also simulate the biochemical composition of the ECM by incorporating bioactive molecules such as growth factors, peptides, or adhesive proteins. These substances serve as cues to regulate cell contact [116]. By incorporating healing-promoting elements like β-estradiol, ADSCs, and alkaline fibroblast growth factor into polyisocyanate hydrogels, it stimulates regeneration of the interface between the hydrogel and the tissue and produces a mild to moderate immune response. This process facilitates the healing of connective tissue and restores the function of tissue support [117]. These molecules can be physically encapsulated within the hydrogel or chemically attached to the polymer matrix. Studies have found that compared to a pure hydrogel scaffold, fibroblasts loaded onto NAC hydrogel scaffolds can successfully attach to the NAC scaffold surface, exhibit typical cell morphology, and develop mature pseudopodia (Fig. 4B), and demonstrate improved cell viability and proliferation (Fig. 4C) [106]. It is worth noting that introducing different bioactive molecules into the hydrogel can elicit different cellular activities. For example, growth factors are known to facilitate cell proliferation and specialization [118], while adhesion peptides are known to modify cell binding and movement within hydrogels [119]. Adding recombinant bovine basic fibroblast growth factor to the hydrogel promoted rapid wound closure with 89.5% cell coverage. This significantly enhances cell proliferation and migration, indicating the great potential of hydrogels in repairing full-thickness abdominal wall defects (Fig. 4D) [79].

Early vascular formation serves as the foundation for tissue repair and regeneration [120,121]. Consequently, the promotion of early blood vessel formation has become a vital target for abdominal wall tissue engineering. ADSCs demonstrated potency by converting into multiple cell types such as adipocytes, osteoblasts, and endothelial cells. Hypoxia-inducible factor-1α (HIF-1α), VEGF, and α-smooth muscle actin (α-SMA) expression were significantly increased in ADSCs under hypoxic conditions (Fig. 4E). Cell transduction experiments by lentiviral vectors (Fig. 4F) revealed that HIF-1α, VEGF, and α-SMA protein and mRNA levels were significantly higher in both transduction groups compared with the control group (empty vector) (Fig. 4G). After the knockdown of HIF-1α with siRNA, HIF-1α protein and mRNA levels were significantly reduced in comparison with the negative control group, and VEGF and α-SMA expression was also reduced (Fig. 4H). Furthermore, after HIF-1α knockdown, the proliferation capacity of the cells could not be further enhanced. Following lentiviral transfection of lenti-HIF-1α and lenti-VEGF in culture for 21 days, the presence of WPB, an endothelial cell marker, was identified in both groups using transmission electron microscopy. This finding confirmed that the endothelial differentiation and early vascularization of ADSCs were induced by modulating the HIF-1α/VEGF pathway (Fig. 4I) [108]. Moreover, the abdominal wall remodeling process requires a constant supply of dynamic biological cues to direct cellular behavior. Cai et al. [122] applied the mercaptoene click reaction to create a biomimetic hydrogel capable of detecting early physical indications of inflammatory triggers, together with chemical signals from chemokines and natural adhesion sites, to facilitate accurate tissue remodeling. To summarize, hydrogels present a beneficial environment for cells to flourish by providing structural support, keeping cells hydrated, enabling the transfer of signaling molecules, and giving signals that encourage cell attachment, growth, and differentiation. Hydrogels have characteristics that make them ideal for repairing abdominal wall defects by encouraging tissue growth and facilitating the healing process.

#### Easy manipulation and application in surgical procedures

**Fig. 5.**
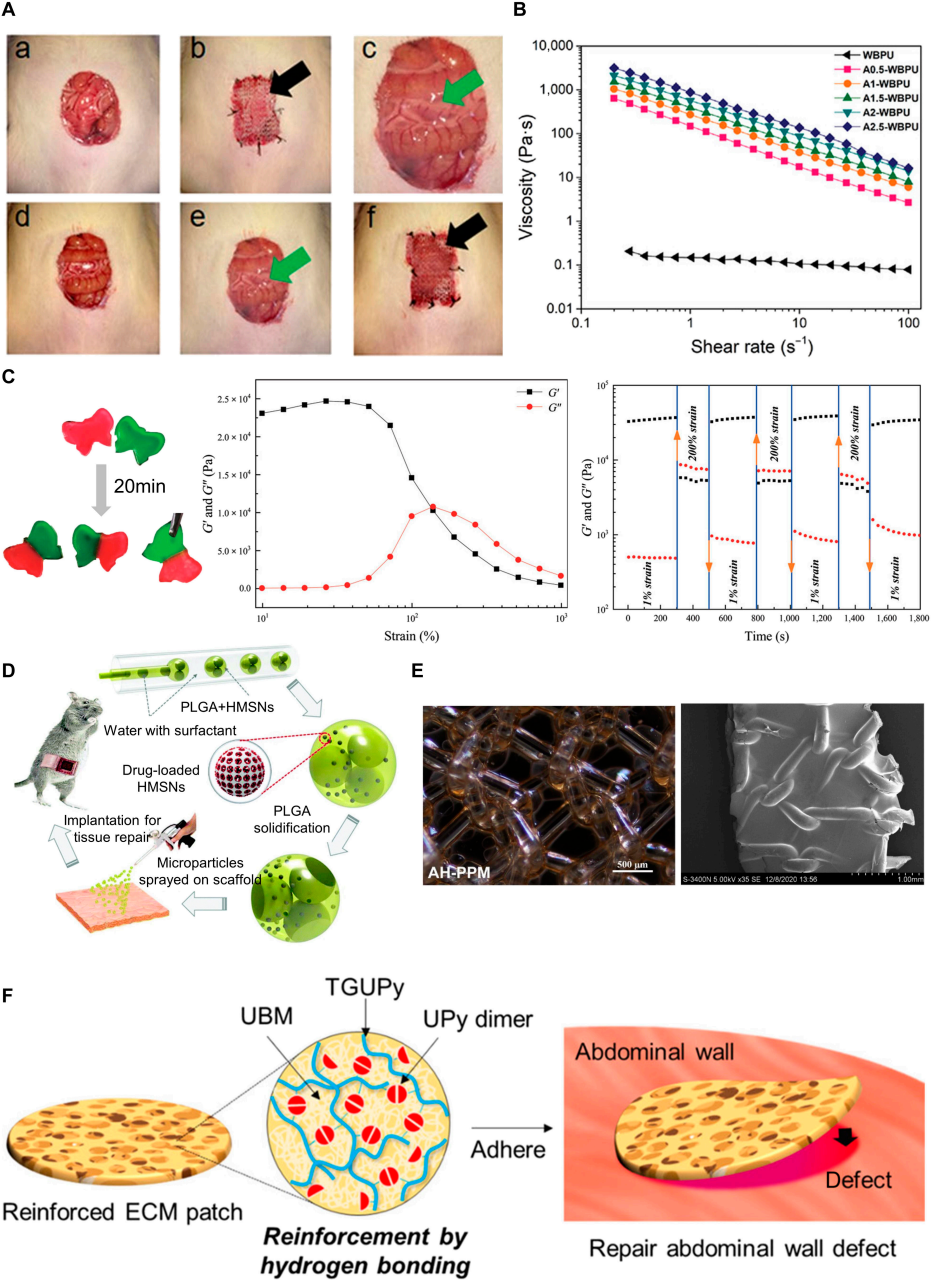
Simplicity of the method using hydrogels. (A) An in situ injection of chitosan-hyaluronic acid hydrogel was applied for abdominal wall repair. Reproduced from [123] with permission from Springer Nature, Copyright 2017. (B) WBPU dispersion and A-WBPU ink flow viscosity profiles according to shear rate increment. Reproduced from [124] with permission from John Wiley and Sons, Copyright 2022. (C) The 4-arm-PEG-CHO/CMCS hydrogel for macroscopic self-repair and rheological recovery testing. Reproduced from [79] with permission from John Wiley and Sons, Copyright 2021. (D) A novel composite scaffold (DFO-HPMs-PADM) was constructed by spraying DFO-loaded HPMs on the surface of PADM. Reproduced from [95] with permission from the Royal Society of Chemistry, Copyright 2018. (E) Optical micrographs and SEM images of AH-PPM. Reproduced from [125] with permission from Elsevier, Copyright 2022. (F) A dECM-based patch adhered to the injured tissue for abdominal wall defect repair. Reproduced from [126] with permission from the American Chemical Society, Copyright 2023.

Hydrogels have become an ideal choice for surgeons due to their ease of handling, injectability, adhesion to tissue surfaces, and ability to provide mechanical support. Hydrogels can be designed with desired viscosity and consistency, enabling surgeons to easily manipulate and apply them. These hydrogels can be formulated in injectable forms, allowing precise and targeted placement within abdominal wall defects using minimally invasive techniques such as syringes or catheters [67]. Deng et al. fabricated a hydrogel with in situ cross-linked CS-HA based on Schiff base reaction, which could be injected in situ to fill abdominal wall defects (Fig. 5A) [123]. Additionally, hydrogels can be engineered to have shear-thinning or self-repairing properties. The viscosity of shear-thinning hydrogels decreases under shear stress, making them easier to inject or diffuse into the tissue (Fig. 5B) [124]. Upon the cessation of shear force, the hydrogels regain their original viscosity, providing stability and support to the repaired tissue after abdominal wall defect closure. The self-repairing ability of hydrogels is dependent on the dynamic interactions among the functional groups present inside them. This means that hydrogels can autonomously change their structure in response to mechanical damage, further enhancing their handling characteristics. For example, in the case of a damaged postoperative incision, a hydrogel with self-healing behavior can be used to prevent patch rupture or cracking (Fig. 5C) [79]. Furthermore, hydrogels can adhere well to tissue surfaces through methods like injection or coating, promoting retention at the site of application on the defect. For example, microgels containing bioactive factors can be directly delivered to abdominal wall defects by injection (Fig. 5D) [95], or hydrogels can be pre-coated onto mesh surfaces to improve mesh performance, reduce foreign body reactions, promote tissue integration, and ultimately aid for abdominal wall repair (Fig. 5E) [125]. Moreover, hydrogels with adhesive properties can better conform to irregular surfaces, ensuring close contact with the abdominal wall defect and preventing leakage or displacement. For example, the physical cross-linking of the urinary bladder matrix (UBM) with supramolecular gelatin aims to prepare a tissue-adhesive decellularized ECM (dECM) patch with superior tissue adherence strength and submerged stability, eliminating the need for sutures and significantly shortening the surgical time to minimize tissue trauma (Fig. 5F) [126]. Furthermore, hydrogels possess excellent biocompatibility, minimizing adverse reactions and inflammatory responses, making them suitable for surgical use. Hydrogels can be designed to be biodegradable, gradually degrading over time to promote tissue regeneration while minimizing the need for subsequent removal. In summary, the ease of handling and application of hydrogels in surgical procedures stems from their injectability, shear-thinning or self-healing behavior, tissue adhesion, and biocompatibility, which contribute to the widespread application of hydrogels in surgical settings.

## Application of Hydrogel for Abdominal Wall Repair

**Fig. 6.**
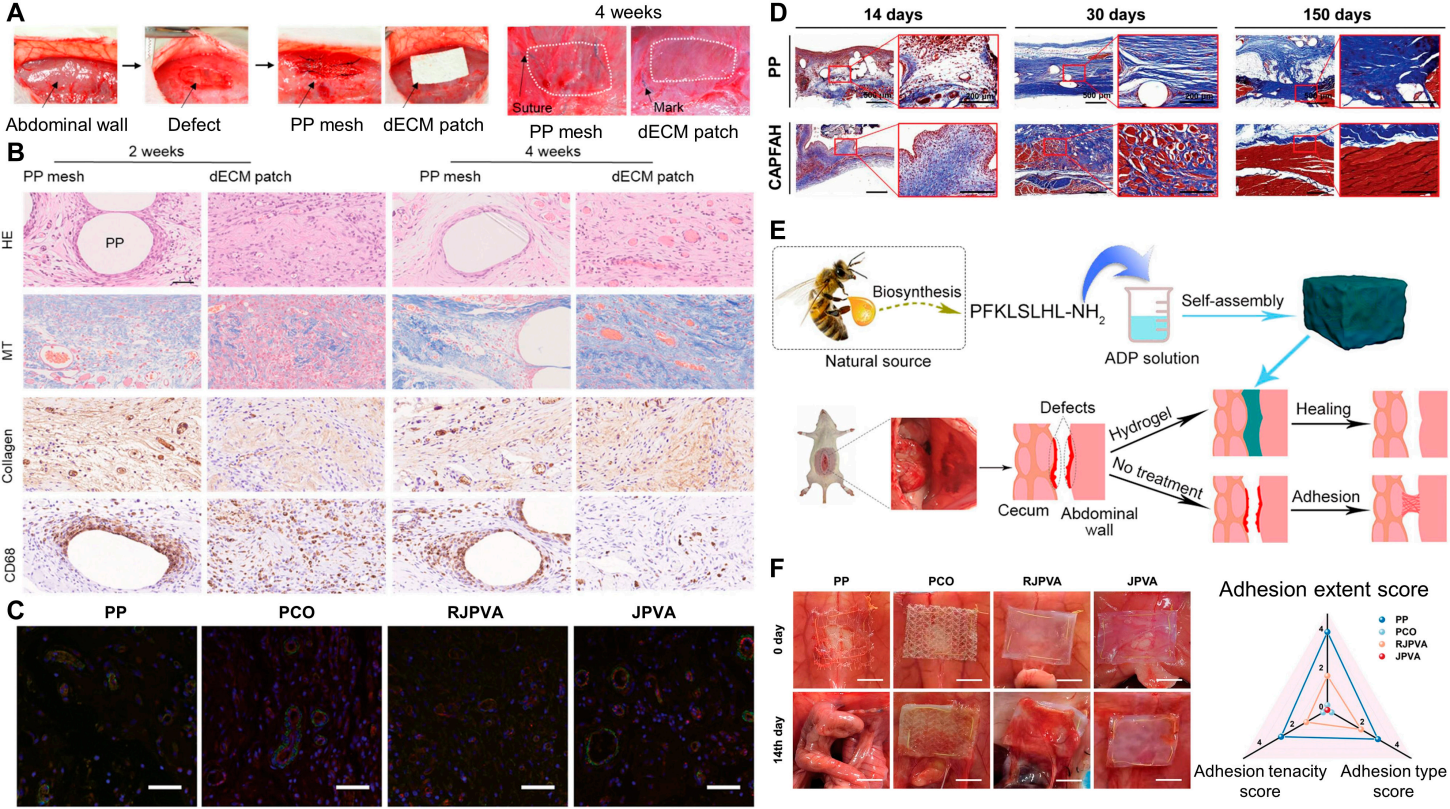
Advances of hydrogel for abdominal wall repair. (A) A rat model was used to evaluate the effect of abdominal wall defect repair. (B) HE, MT, collagen, and anti-CD68 antibody stain of treated abdominal wall defects. Reproduced from [126] with permission from the American Chemical Society, Copyright 2023. (C) Immunofluorescence of CD31/α-SMA of Janus porous hydrogel. Reproduced from [83] with permission from John Wiley and Sons, Copyright 2022. (D) Image of Masson trichrome staining of PP mesh, CAPF, and CAPFAH at 14, 30, and 150 days after implantation. Reproduced from [93] with permission from John Wiley and Sons, Copyright 2023. (E) Self-assembly of natural antimicrobial peptide jelly-1 (J-1) in sodium adenosine diphosphate (ADP) solution to form the J-1-ADP hydrogel for prevention of postoperative adhesions. Reproduced from [139] with permission from the American Chemical Society, Copyright 2022. (F) In vivo prevention of visceral effectiveness of adhesion formation. Reproduced from [83] with permission from John Wiley and Sons, Copyright 2022.

Hydrogel materials have been frequently utilized for repairing abdominal wall defects due to their 5 distinctive properties, leading to advances in this field [127,128]. Animal model studies have shown promising results for various types of hydrogels. Akihiro et al. prepared a UBM-TGUPy (tendon gelatin functionalized with the 2-ureido-4[1H]-pyrimidinone unit) patch based on dECM and verified its biodegradability, mechanical strength, and tissue adhesion promotion effects on the abdominal wall defect model in rats (Fig. 6A). It showed that implanting the UBM-TGUPy patch could induce collagen deposition and blood vessel formation, thereby reconstructing the abdominal defect. There were fewer CD68-positive macrophage aggregates in the group with UBM-TGUPy patches than in the group with non-biodegradable PP mesh, which indicated that biodegradable patches inhibit inflammation and promote tissue repair (Fig. 6B) [126]. Additionally, promoting angiogenesis, enhancing cellular nutrition and oxygen supply, and clearing metabolic waste are crucial steps in abdominal wall repair and regeneration. Therefore, a novel porous hydrogel was successfully developed by Liang et al. and implanted into rat abdominal wall defects. Immunofluorescence staining revealed higher expression levels of the vascular markers CD31 and α-SMA in the JPVA hydrogel group compared with the other groups (Fig. 6C). The histology findings from the research confirmed that the JPVA hydrogel is successful in facilitating abdominal wall repair and enabling substantial regeneration of defects in an experimental model [83]. Long-term assessments are crucial for evaluating the safety, effectiveness, and durability of hydrogel treatments. In a long-term study, staining of the sample tissues was conducted at 14 days, 30 days, and 150 days. At 14 days, the collagen coverage of CAPFAH (CSA/PAM-PPF with adhesive hydrogel) was 90.7% ± 3.2%, far surpassing the healing achieved by the PP mesh. Thirty days after surgery, there was loose collagen present in the group with PP mesh, and there was little to no regenerated muscle observed, indicating poor healing. In contrast, the CAPFAH group exhibited uniformly distributed and well-ordered regenerated muscle. After 150 days of implantation, the PP mesh group still had little muscle formation, while the CAPFAH group showed significantly more ordered and regular morphology of regenerated muscle, similar to the surrounding natural muscle (Fig. 6D) [93]. These results confirm the long-term effectiveness of hydrogel in repairing abdominal wall defects and demonstrate the importance of matching mechanical properties and self-adhesive function with abdominal wall tissue for successful tissue regeneration. Advances in manufacturing technology have allowed for precise control of the structure and properties of hydrogels. Techniques such as 3D bioprinting [129], electrospinning [130,131], microfluidics [132,133], and self-assembly [134] have been used to manufacture hydrogels with complex and layered structures to mimic the natural abdominal wall tissue environment. The utilization of these techniques enables precise positioning of cells and bioactive substances within hydrogels, thereby enhancing their capacity to regenerate tissue for the repair of abdominal wall defects. Furthermore, continuous progress in hydrogel research and development has further expanded its potential. However, prolonged studies are still needed to evaluate the safety, efficacy, and durability of hydrogel interventions to determine their long-term benefits for abdominal wall defect repair and regeneration.

Hydrogel has the potential to effectively address complications associated with abdominal wall defects, including intestinal fistulas, abdominal adhesions, and poor wound repair. Traditional synthetic mesh materials used for implantation are prone to causing intra-abdominal adhesions, while hydrogel materials offer better anti-adhesion properties [135,136]. Intra-abdominal adhesions are believed to be influenced significantly by the presence of infection and bleeding [137,138]. To solve this problem, Zhou et al. proposed a self-assembled hydrogel obtained from the usage of the natural antimicrobial peptide gelatin-1 (J-1) in sodium adenosine diphosphate (ADP) solution, named J-1-ADP hydrogel, to prevent the formation of adhesions in the postoperative period. As a typical amphiphilic antimicrobial peptide with good antimicrobial activity, J-1 can be used for the prevention of tissue infections. As a platelet-activating factor, ADP induces platelet aggregation, activates resting platelets in vivo, and accelerates the coagulation process by inducing platelet aggregation via P2Y1 and P2Y12 receptors, exhibiting highly efficient hemostatic activity. Furthermore, the J-1-ADP hydrogel showed significant anti-adhesion effects in a rat model of sidewall defect-cecum abrasion (Fig. 6E) [139]. Generally, it is difficult to simultaneously achieve anti-adhesion properties of organs and promote defect healing, due to the conflicts between tissue anti-adhesion and adhesion. Inspired by the asymmetric structure of the peritoneum, an anisotropic Janus-structured hydrogel has been proposed, which has a dense structure on the organ-facing side with anti-adhesion properties, and a loose and porous structure on the abdominal wall-facing side, facilitating cell adhesion. An animal model study showed that the ECM-like top surface of one side of the loose porous surface greatly enhanced defect regeneration, whereas the bottom surface of the opposite side of the dense porous surface entirely prevented visceral adhesions. The clinical adhesion score was 0 (Fig. 6F) [83]. Thus, utilizing the flexible and diverse Janus hydrogel method is expected to efficiently address both challenges associated with repairing abdominal wall defects and preventing intra-abdominal adhesions. For secondary intestinal fistulas caused by abdominal wall defects and intra-abdominal adhesions, Qu et al. have innovatively proposed a 4D-printed double-layer hydrogel with responsiveness, flexibility, and biocompatibility based on an acrylamide-acrylic acid/cellulose nanocrystal (AAm-AAc/CNC) network. The 4D bilayer hydrogel completely mimics intestinal curvature and effectively blocks intestinal leakage in an animal model of intestinal fistula [140]. To address poor wound healing caused by abdominal wall defects, Zhao et al. constructed a composite hydrogel scaffold using oxidized branched starch polysaccharide and carboxymethyl CS through a Schiff base reaction. This hydrogel accelerates wound healing associated with abdominal wall defects by promoting macrophage 1–macrophage 2 polarization, reducing inflammation, modulating the inflammatory microenvironment in vivo, and promoting angiogenesis and granulation tissue regeneration [141]. Notably, hydrogels are expected to be the most promising tool for the regenerative medicine field to address complex abdominal wall challenges owing to their significant advantages in addressing abdominal wall defects and their associated complications.

## Conclusions and Prospects

This review outlines the 5 prominent advantages of hydrogels in abdominal wall defect repair and regeneration and highlights the applications and advances in the field. The biocompatibility of hydrogels allows for good integration with host tissues, minimizing adverse reactions. The mechanical characteristics of the hydrogel can be adjusted to meet the specific requirements of the abdominal wall, ensuring optimal support and functionality. Additionally, hydrogels can serve as effective carriers for targeted drug delivery or growth factors, promoting tissue healing and reducing inflammation. By mimicking the biochemical and biomechanical cues present in the natural ECM, hydrogels promote abdominal wall function and tissue structure by providing a favorable microenvironment in which cells adhere, proliferate, and differentiate, and by facilitating the deposition of new tissue. Hydrogels are easily manipulated and employed in surgical operations, making them practical for usage in many medical applications. The flexible properties of hydrogels make them an appealing choice for repairing abdominal wall defect repair. Further study on the connection between hydrogels and abdominal wall defects proves that hydrogels will be a valuable tool in treating such issues clinically.

Although hydrogels have demonstrated innate advantages in abdominal wall defect repair and regeneration, further research and development are still needed to optimize their applications in abdominal wall defect repair and regeneration. Some hydrogels possess excellent biological properties, but their use in load-bearing applications or high mechanical stress regions is limited due to insufficient mechanical strength or stability. Existing toughening strategies have improved the biological performance of hydrogels by incorporating enhancers such as nanofibers, nanoparticles, or biodegradable polymers, but they also weaken the inherent biological properties of hydrogels [142–145]. Enhancing the mechanical characteristics of hydrogels without affecting their biocompatibility is still a challenge. Novel approaches have been recommended to improve the mechanical properties of hydrogels, such as increasing entanglement minus using excessive water, cross-linking agents, or initiators [146], and incorporating force-sensitive components into the hydrogels [147]. Additionally, it is important to properly assess the effects of hydrogel degradation [148,149]. On one hand, the degradation rate is directly related to mechanical support, and on the other hand, the persistent foreign body reaction caused by hydrogel degradation products needs to be addressed [150]. In addition, the uncontrolled release of biologically active substances and cells loaded in hydrogels due to hydrogel degradation can greatly affect the effectiveness of hydrogels in promoting abdominal wall defect repair [151–153]. Moreover, although the use of hydrogels is simpler compared to traditional treatment methods, the majority of studies still focus on combining hydrogels with synthetic mesh [154,155]. One present challenge that needs attention is achieving full replacement of the mesh with hydrogels. Although multiple hydrogels have shown efficacy in repairing abdominal wall defects, it is crucial to evaluate their biocompatibility, stability, and effectiveness during all stages of production, storage, transportation, and application for clinical application. Transitioning hydrogels from the laboratory to clinical application still requires significant advances. With increased biocompatibility, effectiveness, and personalized treatment, hydrogel research will pave the way for a bright future in abdominal wall defect repair.

## Data Availability

Please contact the corresponding authors for data requests.
