# Supplementary material for: Recent Advances in Functional Hydrogel for Repair of Abdominal Wall Defects: A Review
Source: Biomater Res. 2024 Jun 6;28:0031. doi: 10.34133/bmr.0031 (PMC11156463; doi:10.34133/bmr.0031)
Supplement: Supplementary 1 — Figs. S1 and S2 Table S1 [file bmr.0031.f1.pdf]

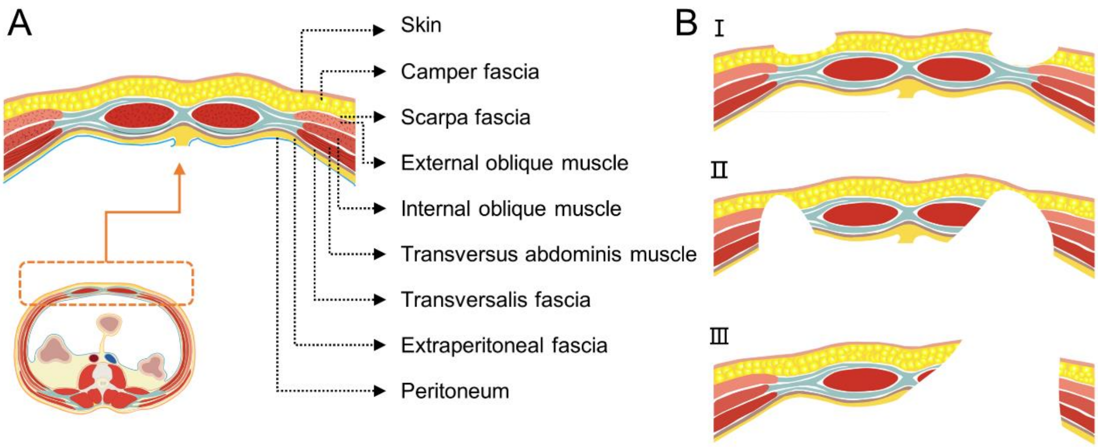

Figure S1. Normal abdominal wall structure and classification of abdominal wall defects. A) Six layers of the abdominal wall from shallow to deep. B) Three types of abdominal wall defects based on the extent of defects. Type I: includes superficial skin and subcutaneous tissue loss. Type II: the abdominal wall myofascial tissue is predominantly absent, but the original abdominal wall skin integrity still exists. Type III: total abdominal wall loss [18,19].

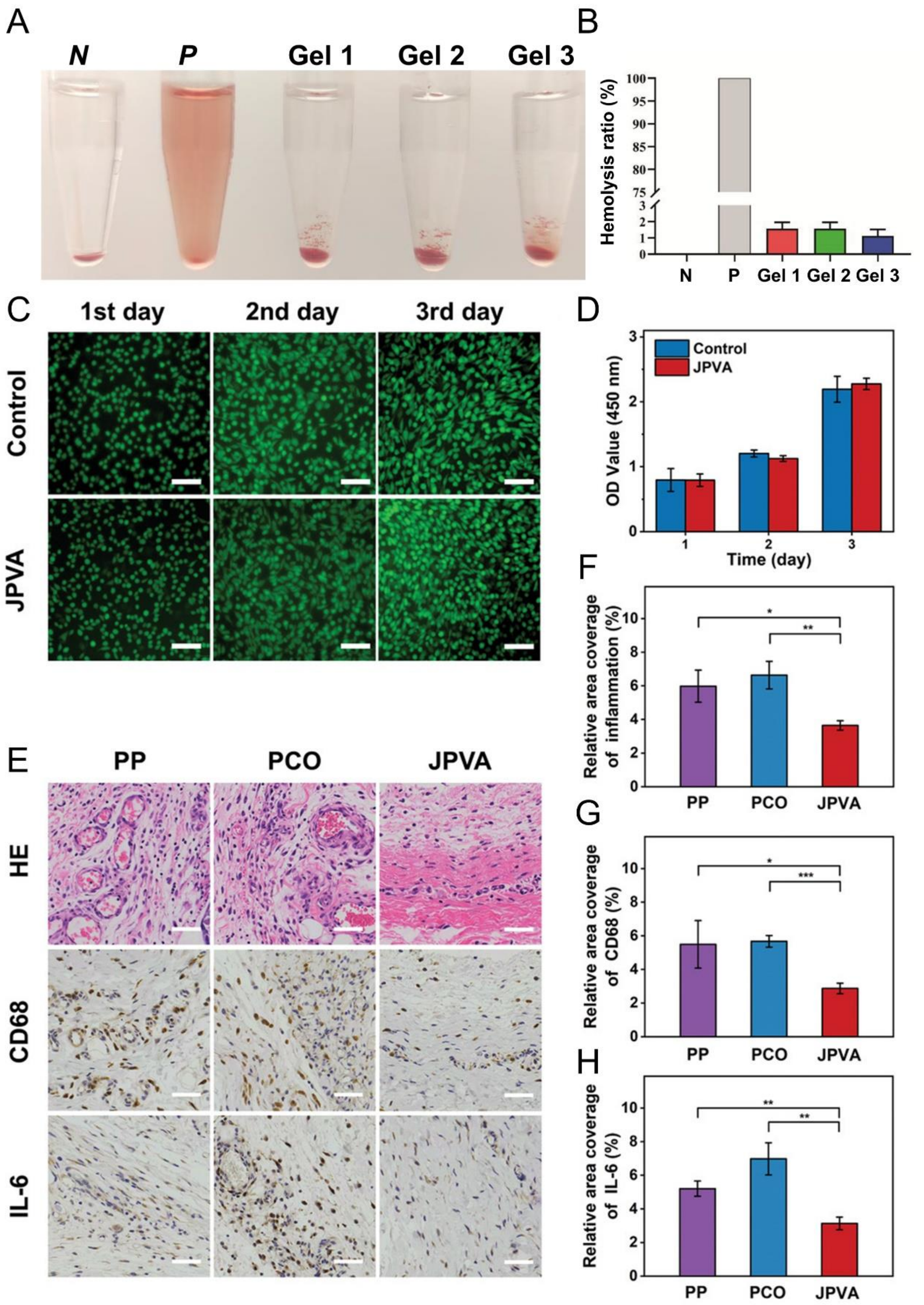

Figure S2. Biocompatibility of hydrogels evaluated in vitro and in vivo. A) Photographs of the hemolytic properties and B) the hemolysis rates of normal saline, distilled water, Gel 1, Gel 2, and Gel 3. Reproduced from [79] with permission from John Wiley and Sons, Copyright 2021. C) The live and

dead staining and D) CCK8 assay results of hydrogels co-cultured with L929 fibroblasts after 1, 2, and 3 days. E) HE staining, immunohistochemical staining for CD68, IL-6 after subcutaneous implantation of PP mesh, PCO mesh, and JPVA hydrogel in vivo. F)-H) Quantitative assessment of inflammatory cells (F), CD68 (G), and IL-6 (H). Reproduced from [83] with permission from John Wiley and Sons, Copyright 2022.

Table S1. The advantages and disadvantages of hydrogel compared to other methods for abdominal wall defect repair.

Methods		Advantages	Disadvantages	Ref.
Hydrogel	1. GT/DA-HA hydrogel<br>2. 4-arm-PEG-CHO/CMCS hydrogel<br>3. PNIPAAm-b-PEG/PLA Scaffold<br>4. CS/HA hydrogel<br>5. AZ-HPCTS hydrogel<br>6. CAPFAH hydrogel<br>7. Pectin-Honey Hydrogel<br>8. JPVA hydrogel<br>9. Silk fibroin hydrogel	1. Biocompatibility<br>2. Tunable mechanical properties<br>3. Drug delivery carrier<br>4. Biomimetic properties<br>5. Ease of use	1. Limited structural support<br>2. Mismatched degradation rate<br>3. Need for combination with other materials (mesh)	[79] [93]<br>[105]<br>[107]<br>[108]<br>[123]<br>[124]<br>[126]<br>[127,128]<br>[139]
Clinical treatment	1. Tension-free patch repair<br>2. Component separation technique<br>3. Abdominal wall expansion technique<br>4. Abdominal compartment reduction technique<br>5. Flap reconstruction	1. Mature technology<br>2. Strong structural support<br>3. High familiarity among doctors	1. High recurrence rate<br>2. High risk of complications<br>3. Limited regenerative capacity<br>4. Complex and demanding surgical procedure	[7] [8]<br>[9] [10]<br>[11,12]<br>[47,48]<br>[49-51]<br>[137]
